# RIG-I-based immunotherapy enhances survival in preclinical AML models and sensitizes AML cells to checkpoint blockade

**DOI:** 10.1038/s41375-019-0639-x

**Published:** 2019-11-18

**Authors:** Michael Ruzicka, Lars M. Koenig, Simone Formisano, Daniel F. R. Boehmer, Binje Vick, Eva-M. Heuer, Hanna Meinl, Lorenz Kocheise, Marcus Zeitlhöfler, Julia Ahlfeld, Sebastian Kobold, Stefan Endres, Marion Subklewe, Peter Duewell, Max Schnurr, Irmela Jeremias, Felix S. Lichtenegger, Simon Rothenfusser

**Affiliations:** 1 Center of Integrated Protein Science Munich (CIPS-M) and Division of Clinical Pharmacology, University Hospital, LMU Munich, Munich, Germany; 2 Einheit für Klinische Pharmakologie (EKLiP), Helmholtz Zentrum München, German Research Center for Environmental Health (HMGU), Neuherberg, Germany; 3 Research Unit Apoptosis in Hematopoietic Stem Cells, Helmholtz Zentrum München, German Research Center for Environmental Health (HMGU), Neuherberg, Germany; 4 Department of Medicine III, University Hospital, LMU Munich, Munich, Germany; 5 Laboratory for Translational Cancer Immunology, Gene Center, LMU Munich, Munich, Germany; 6 German Cancer Consortium (DKTK), partner site Munich, Munich, Germany; 7 Department of Pediatrics, Dr. von Hauner Children’s Hospital, Ludwig Maximilian University, Munich, Germany

**Keywords:** Acute myeloid leukaemia, Cancer immunotherapy, RIG-I-like receptors, Immunological memory

## Abstract

Retinoic acid-inducible gene-I (RIG-I) is a cytoplasmic immune receptor sensing viral RNA. It triggers the release of type I interferons (IFN) and proinflammatory cytokines inducing an adaptive cellular immune response. We investigated the therapeutic potential of systemic RIG-I activation by short 5′-triphosphate-modified RNA (ppp-RNA) for the treatment of acute myeloid leukemia (AML) in the syngeneic murine C1498 AML tumor model. ppp-RNA treatment significantly reduced tumor burden, delayed disease onset and led to complete remission including immunological memory formation in a substantial proportion of animals. Therapy-induced tumor rejection was dependent on CD4^+^ and CD8^+^ T cells, but not on NK or B cells, and relied on intact IFN and mitochondrial antiviral signaling protein (MAVS) signaling in the host. Interestingly, ppp-RNA treatment induced programmed death ligand 1 (PD-L1) expression on AML cells and established therapeutic sensitivity to anti-PD-1 checkpoint blockade in vivo. In immune-reconstituted humanized mice, ppp-RNA treatment reduced the number of patient-derived xenografted (PDX) AML cells in blood and bone marrow while concomitantly enhancing CD3^+^ T cell counts in the respective tissues. Due to its ability to establish a state of full remission and immunological memory, our findings show that ppp-RNA treatment is a promising strategy for the immunotherapy of AML.

## Introduction

Acute myeloid leukemia (AML) is a clonal disease of hematopoietic precursor cells. Despite some improvements in treatment and outcome parameters over the past decades, the prognosis of the disease is still dismal. High risk of relapse despite initial complete remission is the major reason for poor survival rates. This is due to chemorefractory residual leukemic stem cells remaining after intensive chemotherapy [1]. Hematopoietic stem cell transplantation (HSCT) is a potentially curative treatment option based on the graft-versus-leukemia (GvL) effect of allogeneic T cells [2]. However, HSCT is associated with high morbidity and mortality and is thereforee not a treatment option for many  AML patients. Particularly for this patient population, there is a high need for novel therapeutic options including new postremission treatments. Alternative strategies to recruit the immune system for eradication of the disease are studied intensively including antibody-drug conjugates, T cell-recruiting antibody constructs, vaccination concepts, checkpoint inhibitors, and chimeric antigen receptor T cells, although their clinical development for AML still lags behind compared to other malignant diseases [3].

RIG-I-like receptor ligands have been used as a promising strategy for the treatment of solid malignancies including melanoma [4, 5], pancreatic cancer [6] and breast cancer [7] in preclinical models and just recently qualified for a combined phase I/II study in patients with advanced solid tumors (NCT03065023). Serving as a cytoplasmic antiviral pattern recognition receptor, RIG-I senses short double-stranded RNAs with an uncapped 5′-triphosphate moiety (ppp-RNA), a common motif found in viral RNAs [8–12]. Upon binding to viral RNA, RIG-I interacts with the mitochondrial antiviral signaling protein (MAVS) [13–15]. MAVS, once activated, assembles a downstream signaling complex culminating in the activation of nuclear factor kappa-light-chain-enhancer of activated B cells (NF-κB) and interferon regulatory factor 3/7 (IRF3/IRF7), resulting in the release of type I interferons (IFN) and proinflammatory cytokines (reviewed in Chow et al. [16]). Furthermore, ppp-RNA has been shown to induce an immunogenic form of cell death (ICD) in different tumor entities [7, 17–19]. The cytokine release combined with direct sensing of viral RNA by immune cells leads to an adaptive cellular immune response directed against infected cells [4]. By applying an exogenous short ppp-RNA, a viral infection can be mimicked, allowing direction of the immune response towards otherwise altered or potentially harmful targets, such as cancerous cells. While this approach has shown survival benefits in different solid tumor models [4, 6, 7, 20], the treatment efficacy of RIG-I ligands for non-solid tumors in vivo remains elusive. We therefore investigated the potential of ppp-RNA therapy for AML as an example of a hematological malignancy.

RIG-I was first identified as a gene induced by retinoic acid in a promyelocytic leukemia cell line [21] and its expression was described to restrain leukemic activity in AML blasts [22]. Since mainly affecting the bone marrow and blood, we expected AML to be more susceptible to intravenous (i.v.) treatment with ppp-RNA, subsequent systemic cytokine responses and immune cell-mediated cytotoxicity than solid tumors. In addition, we hypothesized that the prominent role of IFN release in ppp-RNA therapy and the subsequent upregulation of programmed death ligand 1 (PD-L1) [23] expression holds potential for combining ppp-RNA with anti-PD-1 receptor antibodies. In recent years, blockade of the inhibitory PD-1/PD-L1 axis resulting in increased cytotoxic activity of T cells was investigated in numerous malignancies with great success [24–27]. However, so far PD-1/PDL1 blockade has only shown moderate success as a monotherapy for AML in clinical settings [3] and in the C1498 mouse model [28]. Here, we explore the responsiveness of AML to ppp-RNA treatment alone and in combination with anti-PD-1 blocking antibodies in the murine syngeneic C1498 AML model and in immune-reconstituted humanized mice with patient-derived xenografted (PDX) AML cells.

## Materials and methods

PDX AML cells were established as described previously [29] and a detailed description of the used mouse models, cell lines, reagents, and methods is provided as part of the supplementary information.

## Results

### Systemically administered ppp-RNA decreases AML burden in vivo, delays AML progression and leads to long-term survival in a murine syngeneic mouse model

**Fig. 1:**
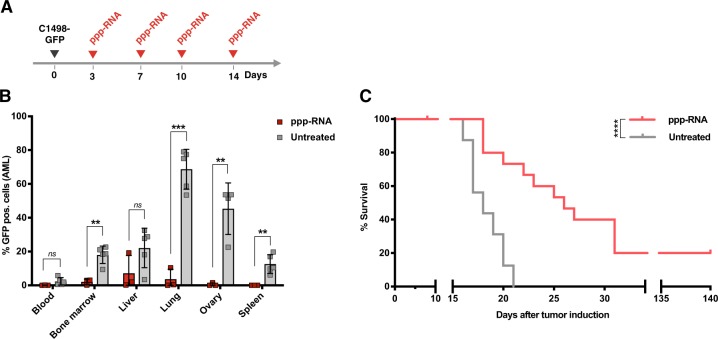
Systemic ppp-RNA treatment in C1498-GFP tumor bearing mice. **a** Therapy scheme for in vivo experiments in the syngeneic C1498-GFP AML model. AML was induced by injecting 10^6^ C1498-GFP AML cells into the tail vein. On days 3, 7, 10 and 14, mice were treated with 50 µg ppp-RNA i.v. **b** ppp-RNA treated (bar charts indicate mean of *n* = 3 with SEM of a single experiment) and untreated (bar charts indicate mean of *n* = 5 with SEM) C57BL/6 mice were sacrificed on day 17. Single cell suspensions of blood, bone marrow, livers, lungs, ovaries, and spleens were analyzed by flow cytometry determining the fraction of GFP^+^ cells (AML cells). Statistical significance was determined using the Student’s *t* test with comparisons indicated by brackets. **c** C1498-GFP AML was induced in C57BL/6 mice (*n* = 16 per group derived from three independent experiments) and ppp-RNA therapy was applied according to the scheme in **a**. Survival data were plotted in a Kaplan–Meier survival curve and statistical significance was calculated with the log-rank test

To asses the in vivo efficacy of RIG-I-based immunotherapy of AML we utilized the C1498 model, a murine AML cell line on C57BL/6 background classified as acute myelomonocytic leukemia [30], which was implanted in immune competent syngeneic mice. It is characterized by highly aggressive growth with regular infiltration of the bone marrow, blood, spleen, liver, lung, and ovaries (data not shown). After inoculation, mice appear asymptomatic for 15–20 days. First signs of disease are commonly followed by rapid progression requiring the sacrifice of the animals. In order to investigate the overall therapeutic potential of systemic ppp-RNA treatment with regard to AML, we inoculated C57BL/6 mice with 1 × 10^6^ GFP expressing C1498 AML cells (C1498-GFP) via tail vein injection. ppp-RNA was complexed with in vivo-jetPEI in order to protect it from nuclease digestion and enable cytoplasmic delivery of ppp-RNA. Fifty micrograms of complexed ppp-RNA were administered intravenously on days 3, 7, 10, and 14 after tumor inoculation (as depicted in Fig. 1a). Mice were sacrificed on day 17, and the tumor burden in various organs was assessed (Fig. 1b). Significant reduction of tumor mass was observed in bone marrow (2.1% vs 18.1% GFP^+^ cells for ppp-RNA treated (*n* = 3) vs untreated (*n* = 5), *p* *=* 0.002), lungs (3.6% vs 68.8% GFP^+^ cells for ppp-RNA treated (*n* = 3) vs untreated mice (*n* = 5), *p* < 0.001), ovaries (0,5% vs 45,3% GFP^+^ cells for ppp-RNA treated (*n* = 3) vs untreated mice (*n* = 4), *p* = 0.004), and spleens (0.03% vs 12.6% GFP^+^ cells for ppp-RNA treated (*n* = 3) vs untreated mice (*n* = 5), *p* = 0.009).

We next evaluated the effects of ppp-RNA treatment on the survival of the mice. C1498-GFP AML-bearing mice were treated as described above and animals were sacrificed at signs of disease such as weight loss, ascites, apathy and/or development of visible tumors and survival data were plotted in a Kaplan–Meier survival curve (Fig. 1c). Beyond a significantly delayed onset of symptomatic AML disease (median survival 26 days vs. 18 days with *p* < 0.0001 for ppp-RNA treated (*n* = 16) versus untreated mice (*n* = 16)), ppp-RNA treatment led to long-term remission in 3 out of 16 (19%) of the treated animals, demonstrating the potential of systemic RIG-I activation in the therapy of AML.

### Treatment efficacy depends on CD4^+^ and CD8^+^ T cells, but not B and NK (natural killer) cells

**Fig. 2:**
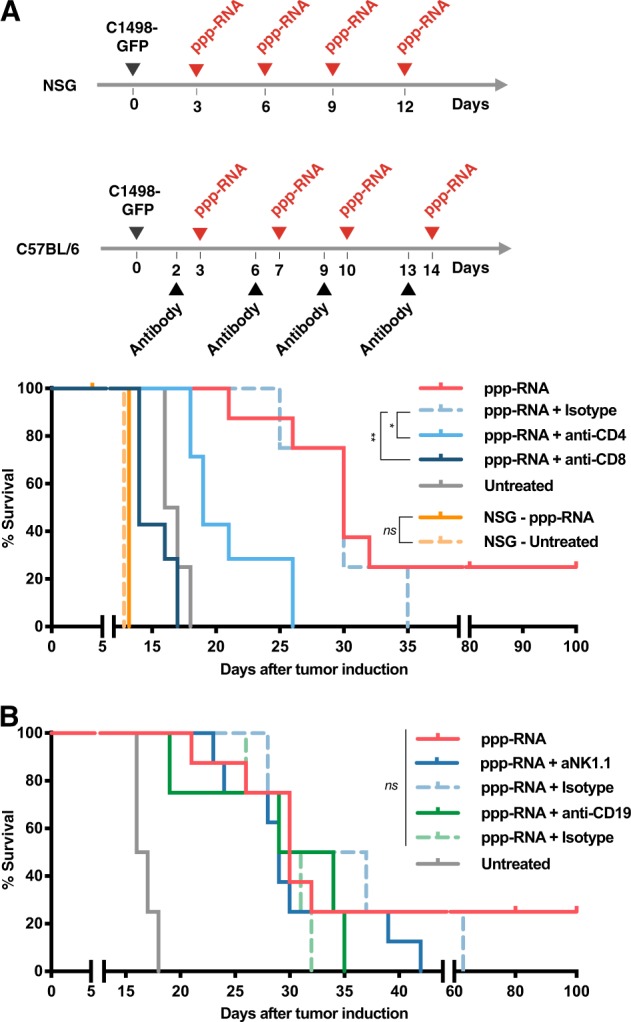
ppp-RNA induced tumor rejection is mediated by cellular immunity. **a** NSG mice and C57BL/6 mice (**a**, **b**) were inoculated with C1498-GFP AML cells and therapy was applied according to the schemes depicted in **a**. NSG mice (*n* = 5 for ppp-RNA treated, *n* = 4 for untreated, both derived from one experiment), C57BL/6 mice (*n* = 8 per group for ppp-RNA treated, untreated and ppp-RNA treated + NK1.1 depleted C57BL/6 mice; *n* = 7 for ppp-RNA treated+ CD4/CD8 depleted C57BL/6 mice. Data derive from two independent experiments; *n* = 4 for ppp-RNA treated + CD19 depleted C57BL/6 mice, these data derive from a single experiment). Depleting antibodies were administered as described in the materials and methods section. Corresponding isotype controls were tested in a total of *n* = 4 mice per group in a single experiment. Survival data were plotted in two Kaplan–Meier survival curves. *P* values of immune cell depleted groups compared to respective isotype controls were calculated using the log-rank test: *p* = 0.018 for CD4, *p* = 0.003 for CD8, *p* = 0.376 for NK1.1, *p* = 0.401 for CD19 depletion

Since the C1498-GFP AML cell line resists transfection with most commercially available RNA transfection reagents in vitro and is thus difficult to target directly with ppp-RNA, it was of great interest to us to narrow down the mechanisms responsible for the observed tumor rejection. In order to discriminate between a direct cytotoxic effect of ppp-RNA on the tumor cells and immune cell-mediated responses, immune incompetent NSG mice lacking T, B, and NK cells were inoculated with C1498-GFP AML cells. ppp-RNA treatment was given on days 3, 6, 9, and 12. Both treated and untreated animals died on day 13 (Fig. 2a) and no significant differences in tumor burden were detected between the two groups via flow cytometry (Supplementary Fig. S1), demonstrating a prominent role of the adaptive immune system in the ppp-RNA-mediated treatment effect.

Based on these findings we next asked which immune cell types were necessary for the survival benefit we observed in treated wild-type mice. Therefore, we specifically depleted CD4^+^, CD8^+^ T cells, CD19^+^ B or NK1.1^+^ NK cells (Supplementary Fig. S2) and compared the treatment success. While CD8^+^ (*p* = 0.003) and CD4^+^ T cell (*p* = 0.018) depletion resulted in significant loss of therapeutic efficacy (Fig. 2a), CD19 (*p* = 0.401) and NK1.1 (*p* = 0.376) depletion remained without significant effect compared to the respective isotype controls (Fig. 2b).

Taken together, these data show that the treatment efficacy of ppp-RNA is dependent on adaptive immunity with CD4^+^ and CD8^+^ T cells being essential mediators of the antitumoral immune response.

### ppp-RNA induced long-term survival in AML-bearing mice depends on systemic MAVS and type I IFN signaling

**Fig. 3:**
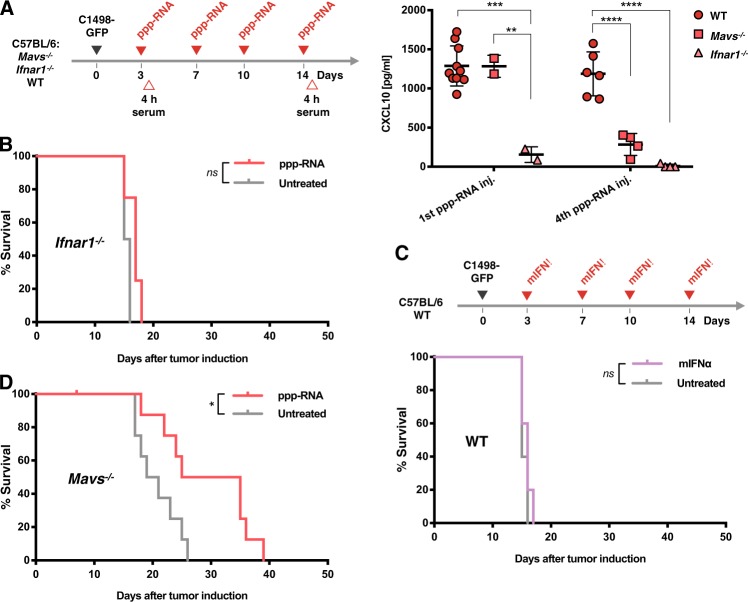
ppp-RNA treatment depends on intact IFN alpha signaling. **a** As depicted in the schematic C57BL/6 WT, *Mavs*^−*/*−^ and *Ifnar1*^−*/*−^ mice were treated with 50 μg of ppp-RNA on days 3, 7, 10, and 14 after inoculation with C1498-GFP AML cells. Blood was drawn after the first (day 3) and fourth (day 14) treatment, and levels of murine CXCL10 were measured via ELISA. Each symbol represents a single mouse and error bars indicate SD. Statistical differences between genotypes at one time point were determined by one-way ANOVA with Tukey’s post-hoc test. **b** *Ifnar1*^−*/*−^ mice (*n* = 4 per group) were inoculated with C1498-GFP AML cells and treated according to the scheme depicted in **a**. Survival data were plotted in a Kaplan–Meier survival curve. *p* = 0.073 for ppp-RNA versus untreated mice. **c** C1498-GFP AML-bearing C57BL/6 wild type mice (*n* = 10) were randomized into two groups, of which one received 5 × 10^4^ IU murine IFN alpha (mIFNα) i.p. on days 3, 7, 10, and 14 as depicted in the schematic in **c**. Survival data were plotted in a Kaplan–Meier survival curve. *p* = 0.352 for mIFNα treated versus untreated mice. **d** C57BL/6 Mavs^−*/*−^ (*n* = 9 per group) were treated with 50 μg of ppp-RNA on days 3, 7, 10, and 14 or left untreated according to the scheme depicted in **a**. Survival data were plotted in a Kaplan–Meier survival curve. The data shown are derived from one (**b**, **c**) or two independent (**d**) experiments

Apart from RIG-I, various other pattern recognition receptors are known to directly or indirectly sense short double-stranded and hairpin RNAs [31], resulting in type I IFN induction. To determine the contribution of off-target effects on the therapeutic outcome of ppp-RNA treatment in the C1498 model and to evaluate the role of RIG-I signaling in the host, we compared the effects of ppp-RNA in wild type (WT), MAVS- and IFNAR1-deficient mice treated according to the scheme depicted in Fig. 3a. To estimate the immunostimulatory activity of ppp-RNA in the respective mice, serum CXCL10 levels were measured as a surrogate marker for type I IFN release after the first and fourth treatment. ppp-RNA therapy in WT and *Mavs*^*−/−*^ mice resulted in comparable serum levels of CXCL10 four hours after the first treatment (*p* = 0.986 for WT versus *Mavs*^−*/*−^ mice; Fig. 3a), while in *Ifnar1*^−*/*−^ mice no CXCL10 was measured after either treatment. Interestingly, CXCL10 levels four hours upon the fourth ppp-RNA treatment in *Mavs*^−*/*−^ mice dropped below detection limit while CXCL10 levels measured in WT mice remained constant (*p* = 0.469 for first vs. fourth treatment in WT mice, *p* = 0.001 for first vs. fourth treatment in *Mavs*^−*/*−^ mice and *p* < 0.001 for WT vs. *Mavs*^−*/*−^ mice after fourth treatment; Fig. 3a).

In *Ifnar1*^*−/−*^ mice, ppp-RNA treatment did not lead to a survival benefit compared to untreated animals (*p* = 0.073; Fig. 3b), indicating a central role of intact type I IFN signaling for the therapeutic effect. We asked the question whether type I IFN may solely mediate the therapeutic effect of ppp-RNA in this particular model of AML. Therefore, therapeutic doses of murine IFN alpha were administered i.p. to C1498-GFP AML tumor bearing WT mice on days 3, 7, 10, and 14. The therapeutic efficacy of ppp-RNA failed to be recapitulated by IFN alpha alone (*p* = 0.352 for IFN alpha treated vs. untreated mice; Fig. 3c), suggesting that type I IFN is essential, but on its own insufficient for the immune response initiated by ppp-RNA treatment.

In *Mavs*^*−/−*^ mice, ppp-RNA therapy prolonged disease-free survival despite disrupted RIG-I signaling (*p* = 0.0193 for ppp-RNA treated vs. untreated mice; Fig. 3d). However, the effect was less pronounced than in WT mice (hazard ratios for ppp-RNA treated vs. untreated mice: 0.229 in *Mavs*^*−/−*^ vs. 0.113 in WT mice). Of note, no long-term survival was observed in *Mavs*^*−/−*^ mice in the treated group.

The results demonstrate that ppp-RNA induced tumor rejection in this AML model is mediated by, but not limited to effects of type I IFN release. Despite CXCL10 levels being comparable after the first ppp-RNA treatment in WT and *Mavs*^*−/−*^ mice, intact RIG-I signaling via MAVS in the host seems to be essential particularly for repeated IFN induction and long-term survival in ppp-RNA treated animals.

### ppp-RNA treatment induces immunological memory

**Fig. 4:**
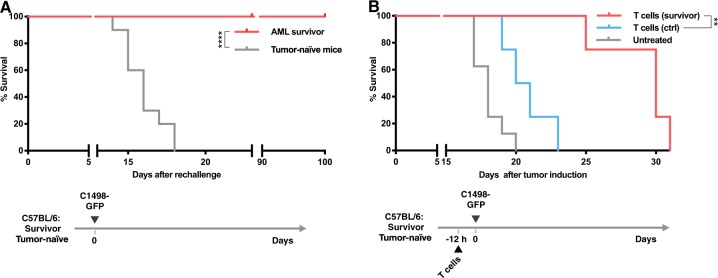
Tumor rechallenge of C1498-GFP AML surviving mice and adoptive transfer of CD8^+^ T cells. **a** C1498-GFP AML surviving C57BL/6 mice (*n* = 7) were rechallenged with 10^6^ C1498-GFP AML cells. No further treatment was applied. Tumor-naive C57BL/6 mice (*n* = 10) served as controls. Survival data derived from three independent experiments were plotted in a Kaplan–Meier survival curve. **b** As depicted in the schematic C57BL/6 mice (*n* = 4) were treated with CD8^+^ T cells from C1498-GFP AML surviving (T cells (survivor), *n* = 3) or tumor-naive mice (T cells (ctrl), *n* = 5), respectively. Untreated mice (*n* = 8) served as controls. C1498-GFP AML was induced 12 h later in all three groups. T cells were isolated as described in the “Materials and methods” section. Survival data derived from two independent experiments were plotted in a Kaplan–Meier survival curve. Significance was calculated using log-rank test

Next, we evaluated if a long-lasting immunological memory was established in ppp-RNA-treated mice that had survived the AML challenge. Surviving mice were rechallenged with C1498-GFP AML cells on day 85–110 after the first AML inoculation and compared to tumor-inoculated control animals. Survivor mice withstood the AML rechallenge in all cases (*n* = 7 for AML surviving vs. *n* = 10 for tumor-naive mice, *p* < 0.0001; Fig. 4a).

In order to further investigate the role of CD8^+^ T cells in the memory responses observed, we adoptively transferred CD8^+^ T cells from survivor mice into therapy-naive WT recipient C57BL/6 mice 12 h prior to inoculation with C1498-GFP AML cells. CD8^+^ T cells from healthy C57BL/6 mice served as control. Significant delay of disease onset was observed in mice treated with CD8^+^ T cells derived from ppp-RNA treated, AML surviving mice compared to tumor-naive donors (*p* = 0.007; Fig. 4b), indicating a central role of CD8^+^ T cells as mediators of the immunological memory established.

### ppp-RNA mediated antitumor response is boosted by PD-1 checkpoint inhibition in vivo

**Fig. 5:**
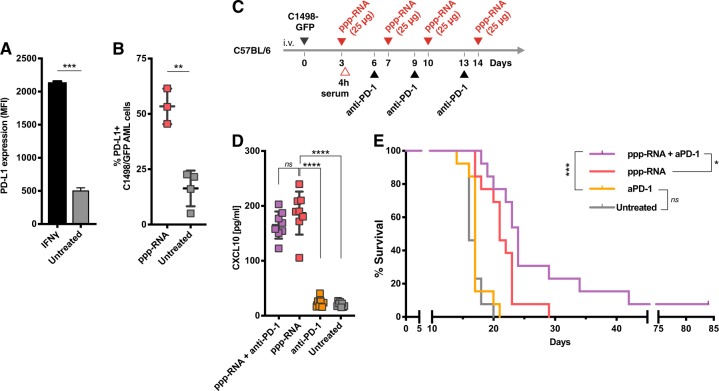
ppp-RNA treatment primes AML cells for anti-PD-1 checkpoint inhibition. **a** 2.5 × 10^5^ C1498-GFP AML cells were seeded in six-well format and treated with interferon gamma. PD-L1 expression was determined by flow cytometry 72 h after stimulation. **b** C1498-GFP AML-bearing C57BL/6 mice received three treatments of 50 μg ppp-RNA on days 8, 11, and 14 after tumor induction. Twelve hours upon the last treatment (day 15), mice were sacrificed and single cell suspensions of lung tissue were analyzed by flow cytometry, determining PD-L1 expression on GFP^+^ AML cells. **c**, **d** C57BL/6 mice (*n* = 13 per group derived from two independent experiments) were inoculated with C1498-GFP AML cells on day 0 and treated with 25 μg of ppp-RNA on days 3, 7, 10, and 14. Hundred micrograms of anti-PD-1 antibody was injected i.p. on days 6, 9, and 13. Levels of murine CXCL10 were determined by ELISA in blood serum obtained 4 h after the first treatment with 25 μg of ppp-RNA (*n* = 8 per group) **c**. Survival data were plotted in a Kaplan–Meier survival curve (**e**). Statistical significance was determined by the Student’s *t* test (**a**, **b**), one-way ANOVA with the Tukey’s post-hoc test (**c**) and the log-rank test (**e**)

In vitro, we observed strong upregulation of PD-L1 on C1498-GFP AML cells upon stimulation with IFN gamma (*p* < 0.001 for IFN gamma treated vs. untreated cells; Fig. 5a). Considering the prominent role of IFN signaling involved in ppp-RNA treatment of C1498-GFP AML (Fig. 3b), we analyzed PD-L1 expression on tumor cells infiltrating lung tissue of C57BL/6 mice, as in this organ we observed the strongest effects of ppp-RNA on tumor load (see Fig. 1b). Mice were sacrificed 12 h after ppp-RNA injection and lung tissue was analyzed by flow cytometry, revealing significant upregulation of PD-L1 on tumor cells (*p* = 0.002; Fig. 5b). Next, we treated C1498-GFP AML-bearing C57BL/6 mice with ppp-RNA on days 3, 7, 10, and 14, applying a suboptimal dose of 25 µg RNA. In addition, 100 μg of anti-PD-1 blocking antibody were administered i.p. on days 6, 9, and 13 (as depicted in Fig. 5c). Serum levels of murine CXCL10 were measured 4 h after the first ppp-RNA treatment on day 3 (Fig. 5d). Treatment with anti-PD-1 antibody had no effect on CXCL10 serum levels. However, the combination of ppp-RNA and anti-PD-1 checkpoint blockade was superior to ppp-RNA or anti-PD-1 antibody alone (*p* = 0.02 and *p* < 0.001, respectively; Fig. 5e) in regard to survival (see also Supplementary Fig. S3A–C). Treatment with the anti-PD-1 antibody only remained without any antitumor effect. Thus, ppp-RNA sensitized AML cells to concomitant checkpoint inhibition leading to superior tumor control. Interestingly, CXCL10 serum levels after the first ppp-RNA treatment (Fig. 5d) correlated with survival time in the ppp-RNA only treatment group (*r* = 0,75; 95% confidence interval: 0.098 to 0.951; *p* = 0,031; *n* = 8) an observation not seen in the group treated with both, ppp-RNA and anti-PD-1.

### Validation of ppp-RNA treatment efficacy in a humanized mouse model of AML

**Fig. 6:**
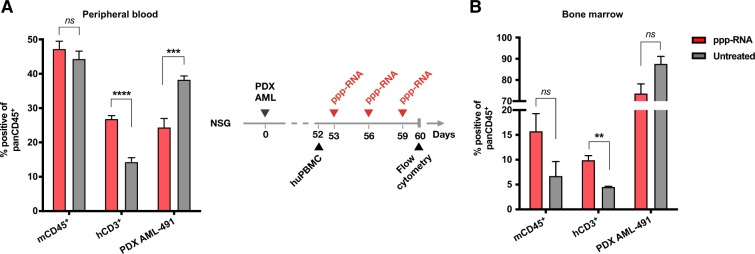
Efficacy of ppp-RNA in a humanized mouse model of AML. Twelve NSG mice were inoculated with 4.5 × 10^5^ PDX AML-491 cells i.v. on day 0 and 10^7^ human PBMCs were injected on day 52. ppp-RNA treatment was given on days 53, 56, and 59. Mice were sacrificed 12 h after the last treatment on day 60. Single cell suspensions of peripheral blood (**a**) and bone marrow (**b**) were analyzed by flow cytometry, determining levels of murine CD45 positive (mCD45^+^) and human CD3 positive cells (hCD3^+^) relating to murine plus human CD45 positive (pan hCD45^+^) cells. Tumor burdens were determined by the detection of mCherry positive cells. Bar charts depict mean values plus SEM of *n* = 6 (**a**) and *n* = 3 (**b**). Statistical significance was determined using a Student’s *t* test with comparisons indicated by brackets

We approached the potential of ppp-RNA-based immunotherapy for clinical translation by testing a genetically diverse panel of five human AML cell lines (MV4-11, OCI-AML3, Molm-13, PL-21 and THP-1) and five patient-derived (PDX) AML blasts (AML-372, AML-388, AML-491, AML-896, AML-981 (see Supplementary Table S1)) for their responses to ppp-RNA ex vivo. These diverse AML cells covering common mutations occurring in human AML all responded to ppp-RNA with the production of CXCL10, the upregulation of MHC-class I, PD-L1 and to variable degrees with the upregulation of FAS and the induction of cell death (see Supplementary Fig. S4). These data confirm that human AML cells have an intact RIG-I signaling pathway and that triggering this pathway induces a measurable but limited direct cytotoxic effect in human AML cells. In addition they suggest that, reminiscent of the effects seen in the C1489 mouse model, ppp-RNA might sensitize human AML cells to T cell-mediated cell death (via enhanced MHC-class I/TCR recognition and Fas/Fas-ligand interaction) and to checkpoint blockade of the PD-1/PD-L1 axis. However, the C1489 model has clearly shown that in vivo the direct cytotoxic effect of ppp-RNA on AML cells alone does not explain the therapeutic benefit of this treatment and that the potential of ppp-RNA treatment can only be seen in the presence of an intact T-cell response. We therefore designed an immune-reconstituted humanized mouse model of AML using PDX AML cells for further validation. NSG mice were inoculated with 4.5 × 10^5^ PDX AML-491 cells via tail vein injection, and tumor growth was monitored via flow cytometry in peripheral blood. An average tumor load of 51% in peripheral blood was detected on day 52 (see Supplementary Fig. S5) and all animals received 1 × 10^7^ human PBMCs from a healthy, partly-HLA-matched donor via tail vein injection. Three doses of 50 μg ppp-RNA were given on days 53, 56, and 59. Mice were sacrificed on day 60 and AML loads as well as immune cell numbers in peripheral blood and bone marrow were determined by flow cytometry (Fig. 6a, b, respectively). Lower tumor burdens were detected in peripheral blood (*p* < 0.001) and as a trend in bone marrow (ns, *p* = 0.071) in ppp-RNA treated compared to untreated mice. Human CD3^+^ T cells were found at higher numbers in the treatment group in both compartments (*p* < 0.0001 for blood, *p* = 0.005 for bone marrow), suggesting enhanced immune cell expansion after treatment.

## Discussion

Targeting RIG-I with ppp-RNA has been described in preclinical studies as a promising strategy in the treatment of various solid tumors [4, 5, 7, 17, 20, 32]. However, no data exist suggesting similar efficacy for hematologic malignancies. Here, we show that systemic activation of RIG-I using ppp-RNA not only extends survival, but also leads to full remission in some mice bearing disseminated syngeneic AML tumors and establishes an immunological memory in surviving animals.

Immunotherapy using ppp-RNA has been shown to rely on two mechanisms, namely a direct cytotoxic effect on the tumor cells [5, 17, 18] and the induction of a cellular antitumoral immune response. While the former mechanism has previously been described as RIG-I-dependent apoptosis induction in a human xenograft melanoma mouse model [17] and different in vitro models, the complete absence of a treatment benefit on disseminated AML tumors in NSG mice indicates no direct cytotoxic or cytostatic effect of ppp-RNA treatment on AML cells in our model. Moreover, the results indicate that the cell-intrinsic antileukemic effect of RIG-I via the AKT and mTOR pathway that has previously been proposed [22] does not play a role in our model. In contrast, rejection of leukemic cells seems to be mediated by the induction of tumor-cell specific cellular immunity, which is consistent with the mode of action of ppp-RNA enhancing cross-presentation by antigen-presenting cells and inducing an adaptive antitumoral response [33]. We further investigated the contribution of individual immune cell types using depleting antibodies in WT mice. We identified CD4^+^ and CD8^+^ T cells to mediate the treatment response while B and NK cells were dispensable. NK cells as mediators of ppp-RNA-induced antitumor responses have been described for B16 melanoma cells [4], but were also dispensable in models of pancreatic cancer [6] and hepatocellular carcinoma (unpublished data), suggesting that the mechanisms of ppp-RNA-induced tumor rejection vary depending on the tumor entity and vulnerability. RIG-I signaling via MAVS in the host plays a crucial role for ppp-RNA treatment outcome. Overall survival in *Mavs*^*−/−*^ mice was inferior to survival in WT mice, while long-term survival and memory formation was observed in WT mice exclusively. The residual effects we observed are possibly linked to Toll-like receptor (TLR) 7, which is known to sense single-stranded RNA and may be activated by ppp-RNA. In fact, previous studies show that repeated systemic activation of TLR7 leads to desensitization of the receptor [34],explaining the diminished CXCL10 induction after repeated treatment with ppp-RNA in *Mavs*^*−/−*^ mice compared to WT mice (Fig. 3a). Nevertheless, the outcome of ppp-RNA treatment has previously been shown to be unimpaired in *Tlr7*^*−/−*^ mice [32], again underlining that MAVS is the critical receptor to achieve long-term remission, independently of potential off-target effects. A central factor of the induction of an antitumoral immune response is type I IFN. Consistent with previously published results, we identified intact IFNAR signaling in the host organism as a key factor for treatment efficacy in the AML model. Disseminated leukemia is known for its inability to induce type I IFN in contrast to subcutaneously grown leukemic tumors, thus failing to activate cellular antitumor immunity [35]. Elevated levels of the interferon-inducible chemokine CXCL10 after treatment suggest that ppp-RNA therapy does induce the release of type I IFN, which in turn paves the way for a cellular immune response as described above. Importantly, IFN treatment alone fails to induce leukemia rejection, emphasizing a ppp-RNA specific effect that initiates an adaptive immune response in a multistep process in which type I IFN plays a critical part [36].

The induction of an immunological long-term memory by ppp-RNA therapy makes this approach particularly interesting for the treatment of AML as it holds potential to prevent relapse. We discovered that all mice surviving C1498-GFP AML after ppp-RNA treatment established an immunological memory, protecting them from a rechallenge with C1498-GFP AML cells. Moreover, CD8^+^ T cells adoptively transferred from AML surviving mice into AML-bearing WT mice extended survival significantly, but could not induce long-term remission. The partial protection is likely explained by the fact that transferred T cells most likely contained only a small fraction of antigen-specific T cells that in addition expanded and worked insufficiently in vivo due to the lack of cognate T helper cells.

Although anti-PD-1 monotherapy proved to be inefficacious in the C1498-GFP AML model, PD-1 inhibition showed augmented therapeutic effects when administered together with ppp-RNA treatment. The combination of both agents was superior in outcome to either of them applied alone, suggesting a priming role of ppp-RNA for the antibody to become therapeutically relevant. This assumption is strengthened by the finding that PD-L1 is strongly upregulated on C1498-GFP tumor cells in vivo after ppp-RNA treatment and was also induced in vitro on human AML cells (Supplementary Fig. S4). As preliminary data indicate, the synergistic nature of ppp-RNA and anti-PD-1 antibodies may hold potential for even higher survival rates if the dose used in our experiments is escalated (see Supplementary Fig. S3). The concept of a priming agent sensitizing leukemic cells to checkpoint inhibition has previously been described using oncolytic viruses [36] and STING agonists [35]. Our results further substantiate the beneficial effect of using an innate receptor agonist triggering an IFN-mediated immune activation and simultaneously blocking IFN-regulated immune checkpoints like the PD-1/PD-L1 axis.

However, even the combination of PD-1 blockade and ppp-RNA treatment induced long-term remissions only in a fraction of mice in our study. A recent publication found a marked immunological heterogeneity even in s.c. tumor models derived from clonal cell lines that explained the differential response to immune checkpoint blockade even in inbred mouse strains [37]. A similar mechanism may explain the differences between responders and nonresponders in the AML model used here, where the disseminated AML cells may reach different niches, in which tumor cells are less susceptible to the treatment by ppp-RNA or the action of specific T cells. In addition, other stochastic processes may influence the establishment of additional immunosuppressive mechanisms other than the PD-1/PDL1 checkpoint. Better transfection reagents reaching those niches, higher doses of ppp-RNA, the combination with other cytotoxic strategies like chemo- or radiotherapy, inclusion of vaccine approaches e.g. based on tumor-mutation specific RNA applications (as described in Sahin et al. [38].) and additional checkpoint inhibitors, e.g. targeting CTLA4, LAG3 or TIM3, might therefore be required to increase the rate of long-term remissions.

To evaluate the potential of ppp-RNA based immunotherapy for clinical translation, we designed a humanized mouse model based on the idea of an allogeneic donor lymphocyte transfusion (DLI). DLI is a procedure that was initially established for relapsed chronic myeloid leukemia after SCT. Recent studies indicate a beneficial effect of DLI in patients suffering from relapsed high-risk AML after SCT [39, 40]. To simulate this setting, we infused human PDX AML-bearing NSG mice with allogeneic human PBMCs. The slow growth of PDX AML cells and looming GvH reactions in this xenograft model forced us to perform the PBMC infusion late, close to terminal stages of the disease and to use low numbers of transfused PBMCs. This left us a very small time window for treatment analysis. Nevertheless, we observed a reduction of tumor mass in the peripheral blood and bone marrow of ppp-RNA treated mice, while at the same time CD3^+^ T cell counts rose in the respective tissues, indicating a boosted GvL reaction triggered by ppp-RNA. More detailed studies will be needed to fully evaluate whether ppp-RNA treatment can potentiate the effects of DLI in relapsed AML patients after SCT or whether the effect is rather mediated by direct cytotoxic effects of ppp-RNA on the leukemic cells.

In summary, ppp-RNA treatment induces an immune cell-mediated response against disseminated AML, leads to long-term survival and establishes an immunological memory protective against leukemia relapse. In addition, treatment with ppp-RNA sensitizes tumors to therapeutic immune checkpoint blockade using an anti-PD-1 antibody.

## Supplementary information

Supplementary figures 1–5

Supplementary methods

Supplementary tables
