# Supplementary material for: RIG-I-based immunotherapy enhances survival in preclinical AML models and sensitizes AML cells to checkpoint blockade
Source: Leukemia. 2019 Nov 18;34(4):1017–26. doi: 10.1038/s41375-019-0639-x (PMC7214254; doi:10.1038/s41375-019-0639-x)
Supplement: Supplementary file 1 — Supplementary figures 1–5 [file 41375_2019_639_MOESM1_ESM.pdf]

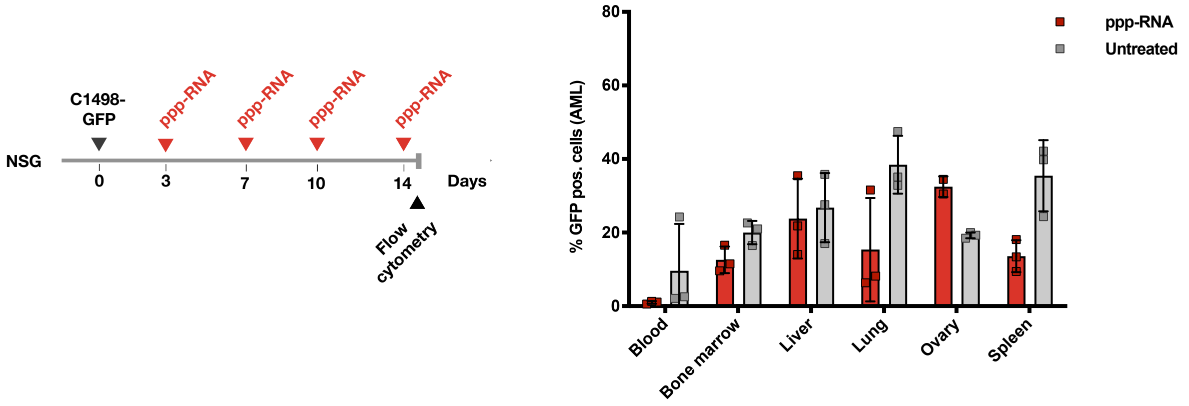

Figure S1. Related to Figure 2. Systemic ppp-RNA treatment in C1498-GFP tumor bearing immunodeficient mice. AML was induced by injecting 10^6 C1498-GFP AML cells into the tail vein of NSG mice. On days 3, 7, 10 and 14, mice were treated with 50 µg ppp-RNA (i.v.) as depicted in the therapy scheme. ppp-RNA-treated and untreated NSG mice were sacrificed on day 14. Single cell suspensions of blood, bone marrow, livers, lungs, ovaries and spleens were analyzed by flow cytometry determining the fraction of GFP-positive cells (AML cells). Data are shown as mean of n = 3 with SEM.

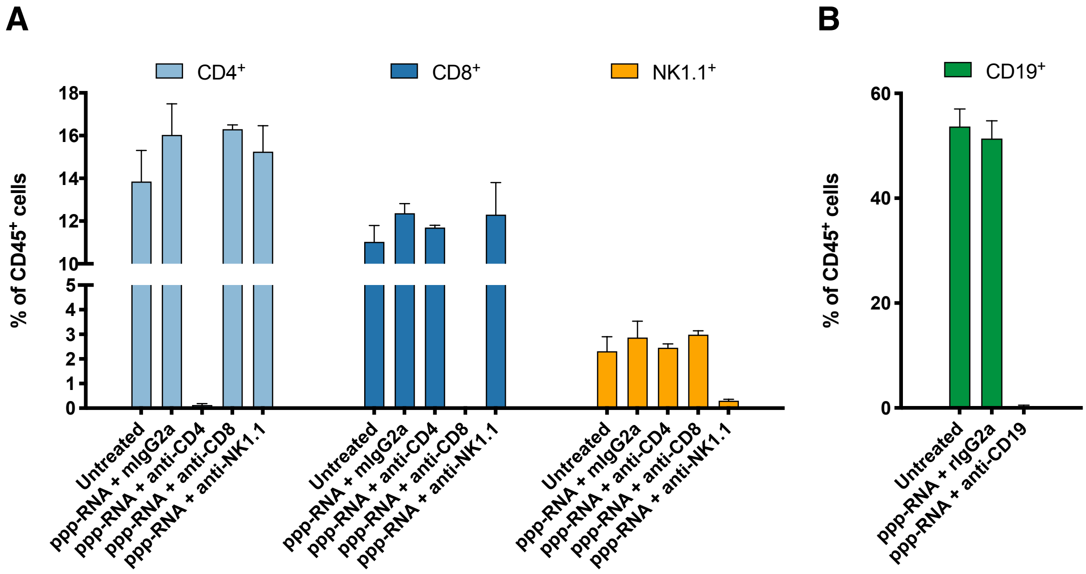

Figure S2. Related to Figure 3. Validation of antibody-mediated immune cell depletion. (A, B) AML was induced by injecting 10^6 C1498-GFP AML cells into the tail vein of C57BL/6 mice. On days 3, 7, 10 and 14, mice were treated with 50 µg ppp-RNA (i.v.). 250 µg of depleting antibodies against immune cell subsets as well as corresponding isotype controls were administered i.p. on days 2, 6, 9 and 13. Blood was withdrawn on day 7 (24 hours after depletion, 1 hour prior to second ppp-RNA treatment) and immune cell depletion was validated by flow cytometry. Data represent the mean + SEM of n = 4 per group for ppp-RNA treated + NK1.1/CD19/rIgG2a depleted C57BL/6 mice, n = 3 for ppp-RNA treated + CD4/CD8/mIgG2a depleted C57BL/6 mice from two independent experiments.

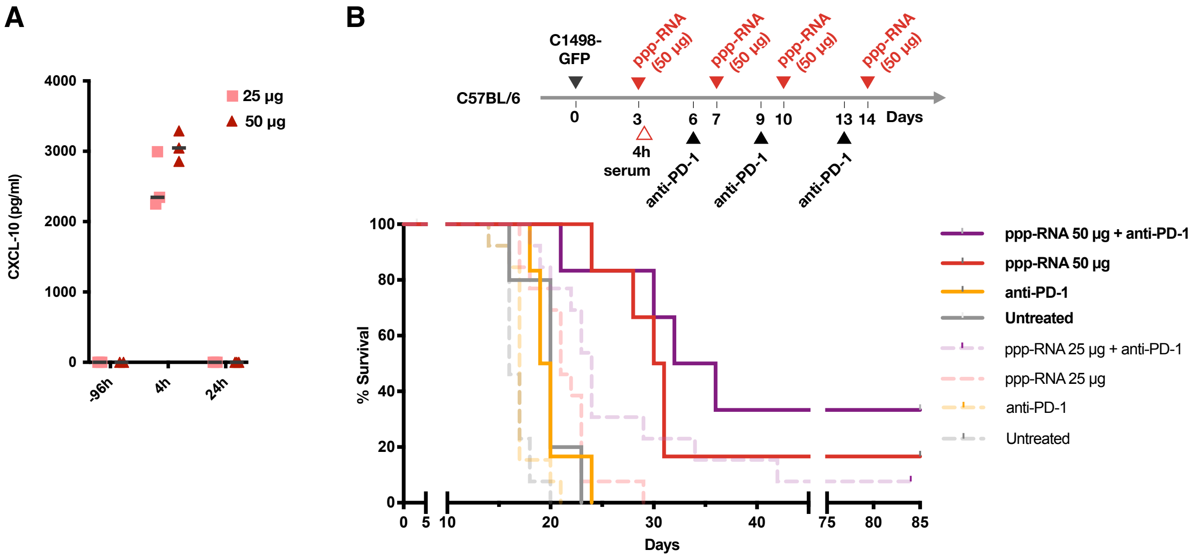

Figure S3. Related to Figure 5. Optimization of dosing improves therapeutic outcome. (A) C57BL/6 WT mice were treated with either 25 *µ*g or 50 *µ*g ppp-RNA and serum CXCL-10 levels were measured before (-96h) or after the treatment (4h and 24h) (n=3). (B) C57BL/6 mice (n = 6 per group; one experiment) were inoculated with C1498-GFP AML cells on day 0 and treated with 50 µg of ppp-RNA on days 3, 7, 10 and 14. 100 µg of anti-PD-1 antibody were injected i.p. on days 6, 9 and 13. Survival data were plotted in a Kaplan-Meier survival curve as an overlay to the data presented in figure 5D (light, dashed lines).

Supplementary Figure S4

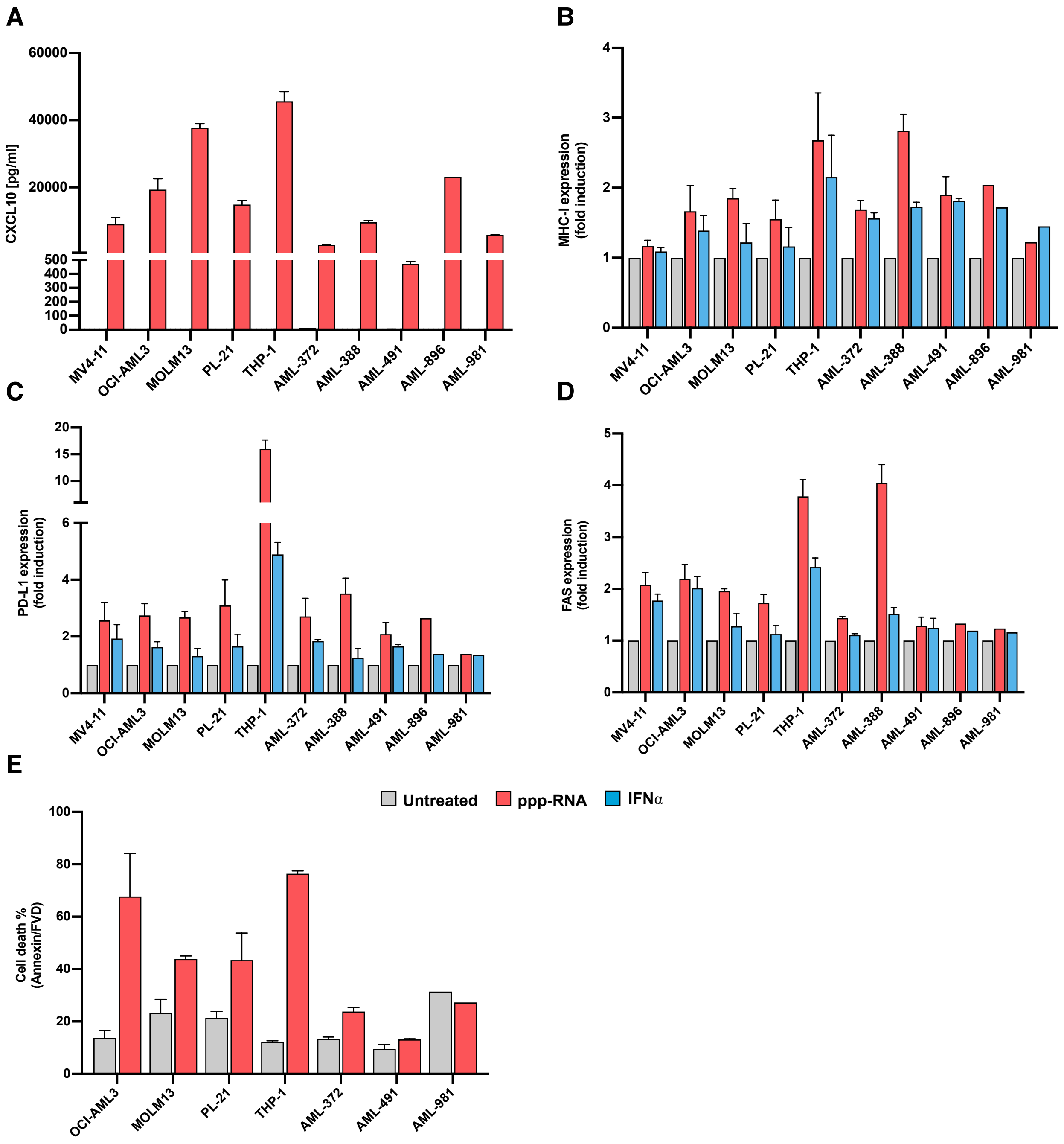

Figure S4. Human AML cell lines were stimulated with ppp-RNA or human IFN-α2a.

1 x 10^5 cells were seeded in triplicates in 96-well plates and left untreated or stimulated with 80 nM of ppp-RNA complexed with lipofectamine RNAiMax or human IFN-α2a (10.000 U/ml). (A) CXCL10 was determined by ELISA in the cell free supernatant of the indicated cell lines either untreated or 24 h after stimulation with ppp-RNA. One representative of at least two independent experiments done in triplicates is shown. Error bars depict the mean +- SEM. (B-D) Flow cytometric analysis of MHC-I (B), PD-L1 (C) and FAS (D) expression 24 h after stimulation with ppp-RNA or human IFN-α2a. Data are shown as fold induction normalized to untreated condition and represent the mean + SEM of one (AML-981, AML-896), two (AML-491, AML-388), three (MV4-11, OCI-AML3, MOLM13, PL-21, THP-1) or four (AML-372) biological replicates each done in triplicates. (E) Flow cytometric analysis of apoptosis induction of a subset of AML cell lines and PDX samples stained with annexin-V and Fixable Viability Dye (FVD) 48 h after stimulation. Data represent mean + SEM of one (AML-981), two (AML-491), three (OCI-AML3, MOLM13, PL-21, THP-1) or four (AML-372) biological replicates each done in triplicates.

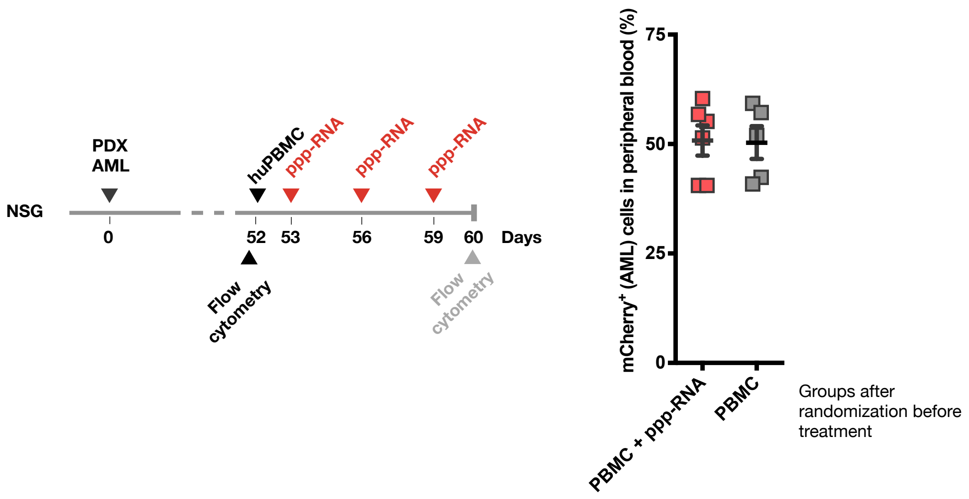

Figure S5. Related to Figure 6. PDX AML burden in NSG mice before human PBMC infusion and ppp-RNA treatment. NSG mice (n=6 per group) were injected with 4.5 x 10^5 PDX AML-491 cells i.v. on day 0. On day 52, blood was withdrawn and the fraction of mCherry-positive cells (PDX AML-491 cells) was determined by flow cytometry to ensure even distribution across the two groups.
