# Supplementary material for: RIG-I-based immunotherapy enhances survival in preclinical AML models and sensitizes AML cells to checkpoint blockade
Source: Leukemia. 2019 Nov 18;34(4):1017–26. doi: 10.1038/s41375-019-0639-x (PMC7214254; doi:10.1038/s41375-019-0639-x)
Supplement: Supplementary file 2 — Supplementary methods [file 41375_2019_639_MOESM2_ESM.pdf]

Supplementary information to:

RIG-I-based immunotherapy enhances survival in preclinical AML models and sensitizes AML cells to checkpoint blockade

Michael Ruzicka*[1], Lars M. Koenig*[1,2], Simone Formisano[1], Daniel F.R. Boehmer[1], Binje Vick[3], Eva-M. Heuer[1], Hanna Meinl[1], Lorenz Kocheise[1], Marcus Zeitlhöfler[1], Julia Ahlfeld[1,2], Sebastian Kobold[1], Stefan Endres[1,2], Marion Subklewe[4-6], Peter Duewell[1], Max Schnurr[1], Irmela Jeremias[3,6,7], Felix S. Lichtenegger*[1,4,5] and Simon Rothenfusser*#[1,2]

* M.R. and L.M.K as well as F.S.L. and S.R. contributed equally to this work.

[1]Center of Integrated Protein Science Munich (CIPS-M) and Division of Clinical Pharmacology, University Hospital, LMU Munich, Germany; [2]Einheit für Klinische Pharmakologie (EKLiP), Helmholtz Zentrum München, German Research Center for Environmental Health (HMGU), Neuherberg, Germany; [3]Research Unit Apoptosis in Hematopoietic Stem Cells, Helmholtz Zentrum München, German Research Center for Environmental Health (HMGU), Neuherberg, Germany; [4]Department of Medicine III, University Hospital, LMU Munich, Germany; [5]Laboratory for Translational Cancer Immunology, Gene Center, LMU Munich, Germany; [6]German Cancer Consortium (DKTK), partner site Munich, Germany; [7]Department of Pediatrics, Dr. von Hauner Children´s Hospital, Ludwig Maximilian University, Munich, Germany

#Corresponding author:

Simon Rothenfusser, Division of Clinical Pharmacology, Department of Internal Medicine IV, University Hospital, LMU Munich, Lindwurmstr. 2a, 80337 Munich.

e-mail: simon.rothenfusser@med.uni-muenchen.de

Tel: +49 (0)89-4400-57301

Supplementary materials and methods

Cells and flow cytometry antibodies

Murine C1498 AML cells were purchased from ATCC (TIB-49TM) and retrovirally transduced with an enhanced green fluorescent protein (eGFP) using a pMX-eGFP plasmid and the PlatE viral producer cell line. Positive cells were sorted via flow cytometry. The human AML cell lines MV4-11, THP-1 and PL-21 were provided by M. Subklewe (University Hospital, LMU Munich, Germany), the human AML cell line OCI-AML3 was provided by K. Spiekermann (University Hospital, LMU Munich, Germany). All cell lines were regularly tested for mycoplasma contamination via PCR or MycoAlert Mycoplasma Detection Kit (Lonza). Authentication of human cell lines by STR DNA profiling analysis was conducted in house.

Patient-derived xenografted (PDX) AML cells were established by serial retransplantation of primary patient leukemic cells in NOD.Cg-*Prkdc^scid^ Il2rg^tm1Wjl^*/SzJ (NSG) mice as described in detail previously[29]. Shortly, patients AML cells were transplanted into 8-12 week old NSG mice. When mice showed clinical signs of illness, mice were sacrificed, and PDX cells were reisolated from bone marrow and/or spleens, and re-injected into new recipient mice for several passages. Furthermore, PDX cells were genetically engineered by lentiviral transduction to express mCherry and enhanced firefly luciferase. PDX cells were applied for repetitive *in vitro* or *in vivo* experiments. PDX cells remained stable over several passages, and resemble the primary tumor cells [1]. Features of the used PDX cells are summarized in supplementary table S1 listing their characteristic mutations and information on the patients they were derived from. Written informed consent was obtained from all patients. The study was performed in accordance with the ethical standards of the responsible committee on human experimentation (written approval by the Research Ethics Boards of the medical faculty

of Ludwig-Maximilians-Universität, Munich, numbers 068-08 and 222-10) and with the Helsinki Declaration of 1975, as revised in 2000. Antibodies used for analyzes by flow cytometry in this study are listed in supplementary table S2 .

ppp-RNA synthesis and transfection

ppp-RNA was synthesized by in vitro transcription (IVT) of a double-stranded DNA template (sense: 5'-*TAATACGACTCACTATA* GCGCTATCCAGCTTACGTAGAGCT CTACGTAAGCTGGATAGCGC-3', antisense: 5'- GCGCTATCCAGCTTACGTAGA GCTCTACGTAAGCTGGATAGCGC *TATAGTGAGTCGTATTA*-3', T7 RNA polymerase promotor sequence is highlighted in italics) using the HiScribe™ T7 Quick High Yield RNA Synthesis Kit (New England Biolabs GmbH, Frankfurt, Germany) according to the manufacturer's protocol. ppp-RNA was purified using the Total RNA Clean-Up and Concentration Kit (Norgen Biotek, Canada). Purity of the product was determined by high pressure liquid chromatography using a DNAPac 200 column. Functionality was tested by measuring human CXCL10 in the supernatant of ppp-RNA transfected 1205Lu melanoma cells (provided by R. Besch, University Hospital, LMU Munich, Germany) by ELISA. For *in vivo* experiments, ppp-RNA was complexed with in vivo-jetPEI (Polyplus-transfection®, Illkirch, France) according to the manufacturer's protocol using a N/P ratio of 6.

Cytokine measurement

For the determination of serum CXCL10, blood of mice was withdrawn 4 hours after treatment with ppp-RNA and serum was analyzed using the Mouse CXCL10/IP-10/CRG-2 DuoSet ELISA (R&D Systems, MN, USA) or Murine IP-10 Standard ABTS ELISA Development Kit (Peprotech Germany, Hamburg, Germany. CXCL10 production by AML cell lines and PDX cells was analyzed 24 h after stimulation with ppp-RNA in the supernatants using the human CXCL10/IP-10 DuoSet ELISA (R&D Systems, MN, USA).

Mice and *in vivo* treatment of AML

In vivo studies were approved by the local regulatory agency (Regierung von Oberbayern, Munich, Germany) and were performed in accordance with the NIH guide for the care and use of laboratory animals. Decisions on the number of animals per group were based on a statistical expert opinion by the The Institute for Medical Information Processing, Biometry, and Epidemiology (IBE), Munich. C57BL/6 mice were purchased from Janvier Labs, France. Mitochondrial antiviral-signaling protein knockout (*Mavs*$^{-/-}$) mice and interferon alpha/beta receptor 1 knockout (*Ifnar1*$^{-/-}$) mice were kindly provided by U. Kalinke (TWINCORE Zentrum für Experimentelle und Klinische Infektionsforschung, Hannover, Germany). At the age of 6 to 8 weeks, tumor inoculation was carried out injecting $1x10^6$ C1498-GFP AML cells intravenously (i.v.) on day 0 into female mice. Mice were randomized before ppp-RNA treatment was given via tail vein injection at a dose of 50 µg on days 3, 7, 10 and 14 if not stated differently. Murine IFN alpha (Miltenyi Biotec GmbH, Bergisch Gladbach, Germany) was administered via i.p. injection on days 3, 7, 10 and 14 at a dose of $5x10^4$ IU. Anti-PD-1 antibody (RMP1-14, Bio X Cell, Lebanon, New Hampshire, USA, Cat.-no. BP0146) and the corresponding isotype control rat IgG2a (2A3, Bio X Cell, Cat.-no. BE0089) were given i.p. on days 6, 9 and 13 at a dose of 100 µg per mouse. NSG mice (Charles River Laboratories, Sulzfeld, Germany) at the age of 5 weeks were inoculated with 4.5×10^5 PDX cells i.v. on day 0 and $1x10^7$ human PBMCs were injected i.v. on day 52. NSG mice were given three i.v. treatments of 50 µg ppp-RNA on days 53, 56 and 59. All mice were monitored daily and sacrificed at signs of affliction or weight loss exceeding 15% in accordance to predefined endpoint criteria. The investigator performed tumor inoculation and treatments. After inoculation, mice were randomized into groups by a blinded subject. Treatment was applied by the investigator without blinding. Mice were monitored and

sacrificed in alternating shifts by neutral scientific and veterinary staff unfamiliar with the objective of the study and the investigator.

In vivo immune cell depletion

Depleting antibodies against CD4 (GK1.5, Bio X Cell, Cat.-no. BP0003-1), CD8a (YTS 169.4, Bio X Cell, Cat.-no. BP0117), CD19 (1D3, Bio X Cell, Cat.-no. BE0150) or NK1.1 (PK136, Bio X Cell, BE0036) were injected intraperitoneally (i.p.) at a dose of 250 µg on days 2, 6, 9 and 13. Rat IgG2b (LTF-2, Bio X Cell, Cat.-no. BE0090) served as an isotype control for CD4 and CD8 antibodies, mouse IgG2a (C1.18.4, Bio X Cell, Cat.-no. BE0085) for the NK1.1 antibody and rat IgG2a (2A3, Bio X Cell, Cat.-no. BE0089) for the CD19 antibody, respectively. Immune cell depletion was validated via flow cytometry using the BD LSRFortessa™ (BD Bioscience, San Jose, CA, USA) 24 hours after antibody injection.

Murine T cell and human PBMC transfers

Murine $CD8^+$ T cells were isolated from spleens. Single cell suspensions were treated with 10 U/ml recombinant human IL-2 (Chiron), 1 μg/ml anti-CD3e (BD Bioscience) and 0.1 μg/ml anti-CD28 (BD Bioscience) antibodies for 24 hours in RPMI containing 10% FBS, 100 IU/ml Penicillin, 100 µg/ml Streptomycin, 2 mM L-Glutamine, 0.5 mM HEPES Buffer (Sigma-Aldrich, Munich, Germany), 1 mM Sodium Pyruvate (Sigma-Aldrich) and 50 µM 2-Mercaptoethanol (Sigma-Aldrich). Consecutively, medium was changed and cells were expanded with 0.5 μg/ml IL-15 (Peprotech). Cells were negatively selected by magnetic cell separation using the $CD8^+$ T Cell Isolation Kit mouse (Miltenyi Biotec). Purity (>98%) was validated via flow cytometry using the BD LSRFortessa™ (BD Bioscience), confirming the T cell phenotype by staining CD3 and CD8. $1x10^7$ $CD8^+$ T cells were injected i.v. into C57BL/6 mice 12 hours prior to inoculation with $1x10^6$ C1498-GFP AML cells. Human peripheral blood mononuclear cells (PBMCs) were extracted

from whole blood by Ficoll density gradient centrifugation and injected into NSG mice via the tail vein at a number of 1×10^7 per mouse.

Statistical analysis

Statistical analyses were performed with GraphPad Prism 7.0c. Data sets were analyzed using (the unpaired) Student's t-test or one-way ANOVA with correction for multiple testing using Tukey's multiple comparison testing as indicated in the figure legends and included a test to determine equal variances within the groups. Overall survival was compared by log-rank test. Hazard ratios stated were calculated applying the Mantel-Haenszel method. n-values reflect total numbers of mice if data derive from pooled experiments. Results were considered statistically significant at values of $p < 0.05$. p values are indicated by *ns* for not significant, * for $p < 0.05$, ** for $p < 0.01$, *** for $p < 0.001$ and **** for $p < 0.0001$. Animals were censored if the cause of death/sacrifice was not AML related or if treatment could not be applied i.v. due to obstruction of the tail vein. Blood samples were excluded from analysis if coagulated. Blood and tissue/organ samples were excluded from analysis if damaged during sample preparation. The criteria for sample exclusion were strictly predefined.

References

1. Vick B, Rothenberg M, Sandhofer N, Carlet M, Finkenzeller C, Krupka C*, et al.* An advanced preclinical mouse model for acute myeloid leukemia using patients' cells of various genetic subgroups and in vivo bioluminescence imaging. *PLoS One* 2015; **10**(3)**:** e0120925.
