# Supplementary material for: RIG-I-based immunotherapy enhances survival in preclinical AML models and sensitizes AML cells to checkpoint blockade
Source: Leukemia. 2019 Nov 18;34(4):1017–26. doi: 10.1038/s41375-019-0639-x (PMC7214254; doi:10.1038/s41375-019-0639-x)
Supplement: Supplementary file 3 — Supplementary tables [file 41375_2019_639_MOESM3_ESM.pdf]

Table S1. Clinical characteristics of AML patients and cell lines

Sample	Disease Stage*	Age[*] [Years]	Sex	Cytogenetics	Mutations[#]
**PDX-Samples**					
**AML-372**	R1	47	f	KMT2A-AF10	BCOR, KRAS
**AML-388**	ID	57	m	KMT2A-AF6	CDKN2A, CEBPZ, KRAS
**AML-491**	R1	53	f	del(7)(q2?1)	BCOR, DNMT3A, ETV6, KRAS, NRAS, PTPN11, RUNX1
**AML-896**	ID	52	f	CN	ASXL2, DNMT3A, FLT3-ITD, IDH2, NPM1
**AML-981**	R1	71	m	CN	ASXL1, DNMT3A, FLT3-ITD, FLT3-TKD, NPM1, TET2
**Cell Lines[§]**					
**MV4-11**		m	10	Hyperdiploid, KMT2A-AFF1	FLT3-ITD
**OCI-AML-3**	ID	m	57	Hyperdiploid	DNMT3A, NPM1
**MOLM-13**	R1	m	20	Hyperdiploid, KMT2A-MLLT3	FLT3-ITD
**PL-21**		m	24	Hypertetraploid	FLT3-ITD
**THP-1**	R1	m	1	Near-Tetraploid, KMT2A-MLLT3	

[*]when the primary AML sample was obtained; [#]targeted resequencing of patient cells and PDX cells, for PDX cells only[1]; [§]Information from DSMZ; ID = initial diagnosis; R1 = 1st relapse; f = female; m = male; del = deletion; CN = normal karyotype

Reference

1 Metzeler KH, Herold T, Rothenberg-Thurley M, Amler S, Sauerland MC, Gorlich D *et al.* Spectrum and prognostic relevance of driver gene mutations in acute myeloid leukemia. *Blood* 2016; **128**: 686–698.

Table S2. FACS antibodies used in this study

Target/Stain	Fluorochrome	Vendor	Catalog Number
anti-human CD3	Pacific Blue	BioLegend	300431
anti-human CD3	FITC	BioLegend	300406
anti-human CD8	APC	Miltenyi Biotec	130110679
anti-human CD33	APC	BioLegend	366605
anti-human CD45	FITC	BioLegend	368508
anti-human PD-L1	PE/Cy7	BioLegend	329717
anti-mouse CD45	Pacific Blue	BioLegend	103126
anti-mouse CD45	PerCP	BioLegend	103130
anti-mouse CD3ε	APC	BioLegend	100312
anti-mouse CD4	PE	BD Bioscience	553730
anti-mouse CD4	PE/Cy7	BioLegend	100528
anti-mouse CD8a	APC	BD Bioscience	553035
anti-mouse CD8	PE	BioLegend	100708
anti-mouse NK1.1	PerCP	BioLegend	108726
anti-mouse CD274 (PD-L1)	PE/Cy7	Biolegend	124314
anti-mouse CD19	PE	BioLegend	115508
anti-mouse CD45	Alexa-Fluor 700	BioLegend	103128
mouse IgG1, κ	APC	BioLegend	400120
mouse IgG1, κ	FITC	BD Bioscience	555748
mouse IgG1, κ	Pacific Blue	BioLegend	400151
mouse IgG2a, κ	APC	BioLegend	400220
mouse IgG2a, κ	PerCP	BioLegend	400256
rat IgG2a, κ	PE	BioLegend	400507
rat IgG2a, κ	PE/Cy7	BioLegend	400522
rat IgG2b, κ	PE/Cy7	BioLegend	400618
rat IgG2b, κ	PerCP	BioLegend	400629
Fixable Viability Dye	eFluor 780	Thermo Fisher Scientific	65-0865-14
To-Pro 3 Iodide		Invitrogen	T3605
Anti-human CD45	BV 785	BioLegend	304048
Anti-human HLA-A, B, C	Alexa-Fluor 488	BioLegend	311413
Anti-human Fas (DX2)	PE/Cy7	BioLegend	305622
Anti-human PD-L1	APC	BioLegend	329708
Annexin V	FITC	Immunotools	31490013
